# Evaluation of a Zulu translation of the Parents’ Evaluation of Developmental Status

**DOI:** 10.4102/phcfm.v9i1.1365

**Published:** 2017-06-28

**Authors:** Maria van der Merwe, Marlé Cilliers, Celéste Maré, Jeannie van der Linde, Mia le Roux

**Affiliations:** 1 Department of Speech-Language Pathology and Audiology, University of Pretoria, South Africa

## Abstract

**Background:**

One of the greatest challenges in early communication intervention in South Africa is developing and implementing successful identification strategies in primary health care (PHC). A shortage of trained PHC personnel is one of the barriers to providing adequate health services in South Africa. This dearth of services creates the need to substitute clinician-administered developmental screening tools with parent-administered tools.

**Aim:**

To determine the accuracy of the Zulu Parents’ Evaluation of Developmental Status (PEDS) in comparison with the outcome of the English PEDS.

**Setting:**

The data were collected in a clinical, non-contrived environment at Stanza Bopape Community Health Clinic in Mamelodi, City of Tshwane.

**Methods:**

The PEDS is a standardised, parent-completed questionnaire regarding the child’s general development. The English PEDS was translated into Zulu by a Zulu linguist. There were 99 potential participants in the study of whom 83 met the necessary prerequisites.

**Results:**

Of the participants whose home language is Zulu, 54% preferred the PEDS in English over the PEDS in Zulu. This indicates a skewed preference towards English, with only slight associations between language preference and age, education and home language.

**Conclusion:**

The Zulu PEDS displayed high positive and negative correspondences, representative of an accurate translation of the English PEDS. It is recommended that this study should be repeated in a community where the majority are Zulu home language speakers.

## Introduction

Five to ten per cent of the paediatric population in South Africa have developmental disabilities.^1^ More specifically, impairment in the acquisition of language is one of the primary markers of developmental delays that may influence academic and social functioning throughout life.^2^ Research has shown that there is a growing relationship between poverty and risk for delays or disabilities.^3^ South Africa has a large number of poverty-stricken communities.^4^ Therefore, there is an increased prevalence of infants that are classified as high risk for disability and communication disorders.^5^ This increased prevalence highlights the fact that early identification of developmental delays should be a primary concern for all health care professionals, as the efficiency of early intervention following identification is largely linked to the age of identification.^6^ The younger the child is when a potential risk is identified, the greater the benefits of intervention that can capitalise on neuro-developmental plasticity.^7^ One of the greatest challenges in early communication intervention is developing and implementing successful identification strategies in primary health care (PHC).^7^

Ideally, developmental screening should be conducted by nurses and other PHC workers,^8^ but 60% of PHC clinics struggle to fill the existing posts. The shortage of trained PHC personnel is one of the barriers to providing adequate health services in South Africa,^9^ and it results in excessive referrals to secondary- and tertiary-level hospitals.^8^ This dearth of services creates the need to substitute clinician-administered developmental screening tools that are time-consuming with parent-administered developmental screening tools.^10^ Parent-administered tools may lighten the caseload of PHC personnel, as the PHC sector is under enormous pressure because of large patient numbers, understaffing and poor resources.^11^

Not only do parent-administered tools lighten the load of health care professionals, but research has indicated that in PHC, tools that depend on information obtained from parents are the most effective.^12^ Professionals may underestimate the capabilities of younger children, especially those with disabilities, whereas parents can give a much more accurate description of their development.^13^

Parent-administered tools provide information across various contexts, and can include behavioural skills that cannot always be observed during formal testing. It is also easier for parents to elicit responses from their own children. Professionals can therefore spend less time assessing the children.^14^ Parent evaluations are also cost-effective, as the screening can be conducted at home, as a web-based assessment or over the phone.^15^ A serious concern in a multilingual environment, however, is the potential language barrier that may result in the tool failing to identify developmental delays.^16^

In multilingual contexts, allied health care professionals need linguistic competence and reliable resources to render effective services.^17^ The lack of linguistic competence and resources results in a gap between the linguistically diverse client populations and the typical linguistic homogeneity of health care professionals.^18^ The linguistic diversity in South Africa with its 11 official languages poses a challenge to professionals in their attempt to provide equitable services to all clients.^19^ A need for linguistic competence and appropriate resources in order to render effective services in South Africa is evident. Contributing to the challenge is the fact that the majority of South African health care professionals received their training in Afrikaans or English only.^20^ Consequently, practices do not reflect the multilingual nature of the population, resulting in numerous challenges that prevent clear communication between therapists and their clients.^21^

According to the 2011 census statistics, South Africa has 51 770 560 inhabitants of which 11 587 374 (22.7%) were first language Zulu speakers.^22^ Rendering services to parents in Zulu may result in improved communication, which subsequently may lead to better therapist–client relationships, a clearer understanding of health and development, and ultimately improved outcomes for services rendered in underserved communities.^23^ A translated developmental screening tool, which serves as an entry level to early intervention, may aid in early identification of infants from Zulu-speaking households.

The translation of tools, on the other hand, may potentially result in less accurate results.^24^ A study concluded that one of the biggest problems translators face when translating from English to African languages, such as Zulu, is the lack of terminology in some of the more specialised fields.^25^ Thus, specialised terminology may be better known and understood in English. For instance, the Western Aphasia Battery (WAB), a standardised English assessment tool used to evaluate speech, language and cognition of neurologically impaired adults, was translated and evaluated in a research study. The study aimed at determining whether the linguistic complexity of the WAB changed after the translation from English to Zulu. The results suggested notable differences in semantics and vocabulary,^24^ which influenced the accuracy of the tool. It was therefore recommended that professionals need to proceed with caution when using translated materials that have not been appropriately and systematically adapted for a different linguistic environment.^24^

In contrast, there are many examples of successful translations of tools into different languages. One such example involves the Parents’ Evaluation of Developmental Status (PEDS). It is an English standardised and validated developmental screening tool that has been translated into many languages including Vietnamese, Chinese and Malay. An example of a successful translation is the Spanish version that was deemed linguistically appropriate for a Latin-American context.^26^

Considering the fact that linguistically appropriate developmental screening tools are needed in South Africa, it may be valuable to translate the PEDS test into Zulu and to evaluate the outcome of the tool, that is, pass or refer, as a means to determine the accuracy of the translated tool. This consideration led to the following research aim: to determine the accuracy of the Zulu PEDS in comparison with the outcome of the English PEDS and to determine the language preference between the English and Zulu PEDS amongst the Zulu population.

## Research methods and design

### Study design

A quantitative, comparative research design was used to compare the outcome of the translated Zulu PEDS and the PEDS in English. In addition, the participants’ language preference between the two tools was determined.

### Setting and study population

The data were collected in a clinical, non-contrived setting, at Stanza Bopape Community Health Clinic in Mamelodi, City of Tshwane. Mamelodi has a population of about 334 577 people speaking a variety of African languages. Of these languages, the most prevalent are Northern Sotho (42.35%) and Zulu (12.15%).^22^

A total of 99 individuals were asked to participate in the study, but only 83 participants met the prerequisites. The inclusion criteria were as follows: firstly, the participants had to be Zulu speakers but could also be fluent in other languages with the ability to read, write and understand Zulu and English. Secondly, the participant had to be a caregiver or parent of a child between the ages of 18 months and 5 years 11 months. Thirdly, the participants must have obtained a minimum education level of Grade 10 so as to ensure that the questions would be read and understood correctly.

The average age of the children in the study was 45.8 months (approximately 3 years 10 months; Standard Deviation/SD = 15.7). The participants were mostly the mothers (*n* = 52, 66%) or the grandmothers (*n* = 18, 23%) of the children. The data concerning level of education showed that 12% (*n* = 9) of the participants had completed Grade 10 and 14% (*n* = 10) Grade 11. The majority of the participants (74%, *n* = 55) had completed Grade 12. Of the participants, 66% (*n* = 50) were younger than 40 years, whereas 34% (*n* = 26) were 41 years old and above.

The majority of the participants were Zulu home language speakers (*n* = 42, 54%); similarly, more than half of the participants’ language of education was Zulu (*n* = 41, 52%). Northern Sotho was also a prevalent home language (*n* = 13, 17%) and language of education (*n* = 17, 21.5%). Although only one participant was an English home language speaker (*n* = 1, 1%), several had attended school in English (*n* = 11, 14%).

### Material and apparatus

The PEDS in English consists of 10 questions regarding a child’s general development. The PEDS that has been translated into Zulu consists of the same 10 questions. These two forms of the PEDS questionnaire were used in the current study.

The responses to the PEDS questionnaires were interpreted using the PEDS interpretation form, which explains the five evidence-based pathways of referrals.^27^ The first pathway, A, constitutes two or more predictive concerns and requires referral to an allied health care professional. Pathway B is followed when one predictive health concern is indicated. The child should be screened for health or sensory problems, and a second developmental screen can be considered. Pathway C includes non-predictive concerns and counselling should be provided in areas of difficulty. Follow-up screening is required. Pathway D should be followed when parents have difficulty communicating their concerns. A second screen that directly elicits the child’s skills can be conducted. Pathway E indicates no parental concerns and the child is perceived as typically developing; thus, it is a low-risk path. Pathways A–D are interpreted as ‘failing the screening’, and pathway E is considered a pass.^27^

A background information questionnaire was given to each participant (caregiver or parent) to complete. The purpose of the questionnaire was to gather background information, including home language and the level of education obtained. In addition, a language preference questionnaire was given to the caregiver or parent to complete, to indicate their language preference between the English and Zulu PEDS. This language preference questionnaire was based on the Short Acculturation Scale.^28^

### Procedures

#### The translation of Parents’ Evaluation of Developmental Status

The English PEDS was translated into Zulu by a linguist. Then, the tool was back-translated to English by another linguist. The reason behind this was to ensure correctness and reliability of the translation, as many Zulu words have different meanings in different contexts.

A panel validated the Zulu translation by comparing it with the standardised English PEDS. The panel comprised a speech-language pathologist academically fluent in Zulu and English, a second lecturer in linguistics fluent in English and Zulu, the supervisors and the researchers of the study. Each word was critically evaluated and, if necessary, changed to guarantee understanding within the context of the question. After the panel discussion, the final changes were made to the Zulu translation.

#### Data collection

Data were obtained over a period of seven days for three to four hours per day. Convenience sampling was used to select participants for the study. Parents or caregivers waiting at the clinic for services such as immunisations, medical consultations or at the pharmacy were asked to participate in the study. The consent form was fully explained prior to the completion of the questionnaires.

The randomisation of questionnaires was chosen as the sampling method to prevent a learning effect. Each questionnaire and its corresponding participant were allocated a number. The odd-numbered participants were given the English PEDS first and the Zulu PEDS thereafter. All the even-numbered participants received the Zulu PEDS first. After completing both questionnaires, the participants were asked to indicate their language preference.

#### Data interpretation and analysis

Two different interpretations (outcomes) were used to pass or refer the child of the participant. Outcome 1 was path A–D, indicating the presence of predictive or non-predictive concerns, and therefore a second screening or referral was required. Outcome 2 consists of paths A and B – indicative of one or more predictive concern that should lead to a referral of the child.

Analyses were completed using Stata 12.1.^29^ Percentages were used to describe the data. Pivot tables were used to illustrate the pass and referral rate of the English and Zulu versions. Overall outcomes of the paired difference tests, between the Zulu and English questions, were assessed by means of the non-parametric Wilcoxon signed-rank test. A significance level of *p* ≤ 0.05 was used. Pivot tables were used to determine the positive correspondence (i.e. the proportion of positive screen outcomes correctly identified) as well as the negative correspondence (i.e. the proportion of negative screen outcomes correctly identified). The relationship between the language preference for PEDS, the education level and the age of the participant were evaluated by means of a chi-squared test and Spearman’s rank correlation coefficients.

#### Ethical consideration

Ethical clearance (permit number: 102/2011) was obtained from the research committee of the Faculty of Humanities, University of Pretoria, Faculty of Health Sciences, University of Pretoria, and the Tshwane District Department of Health research committee. This article is presently not under consideration at another publication nor will be while it is under consideration with the African Journal of Primary Health Care and Family Medicine.

## Results

According to the English PEDS (see outcome 1, Table 1), 66% (*n* = 54) of the participants required an additional screening or referral. The Zulu PEDS yielded similar results with 61% (*n* = 50) of the participants. With outcome 2, fewer referrals are seen for both the English and the Zulu PEDS, 50% (*n* = 41) and 45% (*n* = 37), respectively. A small difference can be noted between the referral rates of the English and the Zulu PEDS.

**TABLE 1:** Pass and fail distribution of the English PEDS and Zulu PEDS.

Variable	English PEDS outcome 1†	English PEDS outcome 2‡	Zulu PEDS outcome 1†	Zulu PEDS outcome 2‡
Pass rate	34% (28/82)	50% (41/82)	39% (32/82)	55% (45/82)
Referral rate	66% (54/82)	50% (41/82)	61% (50/82)	45% (37/82)

PEDS, Parents’ Evaluation of Developmental Status.

† Outcome 1: Path A – D as fail.

‡ Outcome 2: Path A + B as fail.

Because the English PEDS questionnaire has been internationally recognised and validated, it was used as the reference standard test to determine the positive correspondence and negative correspondence of the Zulu PEDS.

Positive correspondence for outcome 1 was determined as 85% and negative correspondence as 86% (see Table 2). The positive predictive value (PPV) for outcome 1, the ability that the Zulu PEDS has to correctly identify referrals, was 92%. The Zulu PEDS’ ability to correctly identify non-referrals, negative predictive value (NPV), was lower at 75%.

**TABLE 2:** Performance of Zulu PEDS.

Variable	Outcome 1†	Outcome 2‡
Positive correspondence	85%	78%
Negative correspondence	86%	88%
Positive predictive value	92% (46/50)	86% (32/37)
Negative predictive value	75% (24/32)	80% (36/45)

PEDS, Parents’ Evaluation of Developmental Status.

† Outcome 1: Path A – D as fail.

‡ Outcome 2: Path A + B as fail.

Outcome 2 yielded similar results, also with high agreement between the English and Zulu responses. Positive correspondence, however, is lower than for outcome 1, with only 78% of the true cases identified by the Zulu PEDS. Negative correspondence for outcome 2 was high, with the Zulu PEDS able to correctly identify 88% of the English PEDS non-referral cases. The PPV and NPV for outcome 2 confirm that the Zulu PEDS identified 86% of referrals and 80% of non-referrals accurately.

A weighted Kappa statistic provided further insight into the level of agreement between the English and Zulu responses. A Kappa statistic of 0.69 (85% agreement) for outcome 1 and 0.66 (83% agreement) for outcome 2 confirmed the high level of agreement between the English and Zulu PEDS results. No significant differences between the responses for English and Zulu PEDS in question 2 and questions 4–9, as well as the overall scores for outcomes 1 and 2, were noted. The only question with a significant difference between the English and Zulu PEDS responses was question 3 (*p* = 0.032). This question evaluates parental concerns regarding their child’s receptive language.

The PEDS in English was preferred by 72% (*n* = 34) of the participants, aged 18–40 years, with the remaining 28% (*n* = 13) participants indicating their preference for the PEDS in Zulu. The majority (60%, *n* = 15) of participants, aged 41–70 years, preferred the PEDS in English and 40% preferred the Zulu PEDS. Although the correlation is not significant, there is a higher percentage for Zulu preference in the older age group than in the younger age group.

The majority of the participants with Grade 12 and a higher education level (74%; *n* = 55) preferred the English PEDS (73%, *n* = 40) to the Zulu PEDS (27%, *n* = 15). In contrast, 44% (*n* = 4) of the participants who left the education system after Grade 10 (12%, *n* = 9) preferred the English PEDS, while 56% (*n* = 5) indicated that they prefer the Zulu PEDS.

No correlation was evident between the participants’ home language and their language preference. Although all of the participants (*n* = 83) were proficient in both Zulu and English, many different home languages were indicated. Of the participants whose home language is Zulu, 46% (*n* = 18) preferred the PEDS in Zulu, whereas 54% (*n* = 21) preferred the PEDS in English. Two participants with Zulu as their home language indicated no specific preference. Of the 13 participants whose home language is Northern Sotho, 85% (*n* = 11) preferred the PEDS in English. One of these participants did, however, prefer the PEDS in Zulu. Of the remaining 22 participants who indicated other home languages, 5% (*n* = 4) preferred the PEDS in Zulu and 23% (*n* = 18) preferred the PEDS in English.

## Discussion

As seen from the results, both the English and Zulu PEDS have a high referral rate. The high referral rate can be attributed to the fact that the study was conducted in a population at risk for developmental delays,^30^ as children raised in low-income families are more vulnerable to these delays.^31^ Studies conducted in other at-risk communities also indicated high referral rates.^30^

Looking at both PEDS interpretations, referral rate for outcome 2 was lower than for outcome 1. This can be attributed to the fact that outcome 1 includes predictive and non-predictive concerns. According to the PEDS score form, from 18 months to 4.5 years of age, behaviour, socio-emotional, fine-motor, self-help and academic parental concerns are considered to be non-predictive domains. Non-predictive concerns can be described as concerns regarding skills that are not perceived as indicative of developmental delay or disability.^27^ It can therefore be deduced that the decrease in referral rate from outcome 1 to 2 is a result of the omission of the domains classified as non-predictive concerns in outcome 2.

A slight difference between the pass and referral rate of the English and Zulu PEDS was noted, with a referral rate of 50% for the English and 45% for the Zulu PEDS. This suggests different interpretations and understanding of the same questions in the two different languages, resulting in different outcomes. When the translation of the WAB^24^ from English to Zulu showed similar differences, the researchers attributed these differences to dialect and not to inaccurate translation. The same may be the case here. The outcomes could have been influenced by the dialectical differences in Zulu that exist between the more colloquial language used in the community and the academic language used in the translation.^24^ A strength of the current study, however, is the accuracy of the translation that is reflected in the high correspondence between the outcomes of the two tools. Although good agreement was achieved, adaptation of the Zulu PEDS may be necessary if contradicting results are yielded in contexts where Zulu is the most prevalent language. More specifically, word selections may need to be evaluated as different cultural and linguistic groups interpret the questions regarding ‘concerns’ differently.^27^ Future research should also consider evaluating the use of bilingual versions of the PEDS tools, as a means to improve comprehension of questions.

For both outcomes 1 and 2, the positive correspondence was high at 85% and 78%, respectively. This suggests that the Zulu PEDS was less sensitive at identifying true referral cases for outcome 2. A contrasting result can be seen with regard to negative correspondence of the Zulu PEDS, as 88% of the non-referral cases were correctly identified, suggesting that the Zulu PEDS was more specific in identifying cases for outcome 2. As anticipated, the negative correspondence for outcomes 1 (86%) and 2 (88%) is high.

The PEDS has been translated into numerous other languages and studies were also completed to determine the positive correspondence and negative correspondence of these translated versions. The positive correspondence of the translations into both Malay (86%) and Chinese (82%)^32^ indicated similar results to the positive correspondence of the Zulu PEDS. The negative correspondence of the Zulu PEDS was considerably higher than that of the Malay (60%) and Chinese (54%) versions.

Inferences can be made regarding the relationship between the age and the language preference of the participants. The division of the age groups showed skewed preference towards the English PEDS (72%) for the younger age group. An increase in the percentage of Zulu preference was seen in the older age group (40%). Further research will be required; however, from this study, it can be deduced that the older the participant, the greater the preference for the PEDS in their Zulu home language. Research has found indications that communities with native home languages are threatened because of a decline in intergenerational continuity, with fewer native home language users in every new generation.^33^ Yet, another study confirms that the lack of native languages in schools results in the younger generations losing proficiency in reading and writing abilities in their native home languages.^34^

The level of education yielded interesting results although, once again, a larger study would be beneficial. Of the participants with an education level of Grade 12 and higher, 73% favoured the English PEDS. In contrast to this, a preference of 44% was seen amongst participants with an education level of Grade 10; thus, the higher the level of education, the greater the preference for the English PEDS. This observation coincides with reports from the previous decade that 80% of education in schools throughout the country occurred in English.^35^ Furthermore, enrolment in African languages across all South African universities declined from 25 000 to 3000 in a short duration of four years because of the view that English is the economically dominant language.^36^ The same factors may still be operative.

From the data analysis, no significant association between the home language of the participants and their language preference was evident, as 54% Zulu home language speakers preferred the English PEDS. A worldwide trend towards English monolingualism with a language shift from the African languages – especially in the Black urban communities – to English was observed some years ago.^33^ This trend may be reflected in the results of the Northern Sotho home language participants as 85% also preferred the English PEDS. The results of this study suggest that African language speakers may prefer English language developmental screening tools. However, a limitation of the current study is that it was carried out in an urban setting only. The results may differ when performed in a rural context; therefore, it is recommended that the study is duplicated in a rural environment.

## Conclusion

The aim of the study was to compare the outcome of the PEDS in English and Zulu. The Zulu PEDS indicated high positive and negative correspondence, representative of an accurate translation of the English PEDS. The results of the study indicated a skewed preference towards English with only slight associations between language preference and age, education level and home language. In order to improve the results, the study should be limited to only Zulu and English home language speakers. This can be achieved through completing the study in other parts of the country, in communities such as KwaZulu-Natal, where 78% of South Africa’s Zulu speakers can be found.
